# Production of monoclonal antibody, Anti-CD3 by hybridoma cells cultivated in Basket Spinner under free and immobilized conditions

**DOI:** 10.1186/1753-6561-7-S6-P57

**Published:** 2013-12-04

**Authors:** Elsayed A Elsayed, Hoda Omar, Hasnaa R Shahin, Hamida Abou-Shleib, Maha El-Demellawy, Mohammad Wadaan, Hesham A El-Enshasy

**Affiliations:** 1 Bioproducts Research Chair, Zoology Department, Faculty of Science, King Saud University, 11451 Riyadh, Kingdom of Saudi Arabia; 2 Natural & Microbial Products Department, National Research Centre, Dokki, Cairo, Egypt; 3 Microbiology Department, Faculty of Pharmacy, Alexandria University, Egypt; 4 City for Scientific Research and Technology Applications, New Burg Al Arab, Alexandria, Egypt; 5 Institute of Bioproducts Development, Universiti Teknologi Malaysia, Skudai, Johor, Malaysia

## Background

Monoclonal antibodies (Mabs) have been recently used for the treatment of virtually every debilitating disease. Packed-bed bioreactors have been used for the cultivation and production of a wide range of cell lines and biologics including MAbs. The principle behind a Packed-bed bioreactor is that the cells are being immobilized within a suitable stationary matrix which is represented by the bed. Packed-bed bioreactors also have the advantage of being capable of generating high cell densities with a low concentration of free cells in suspension; hence, simplifying downstream processing. The present work was designed to compare between the cultivation of hybridoma cells as well as the production of OKT3 MAb in free and immobilized culture conditions.

## Materials and methods

Hybridoma cell line (OKT3), producing IgG2a monoclonal antibodies against CD3 antigen of human T lymphocyte cells were adapted to grow in serum free medium. The specificity of the produced MAbs was determined through the use of indirect immunofluorescence staining of T lymphocytes from peripheral blood followed by flowcytometeric analysis using cell quest software and FACSCalibur. The MAb was continuously produced by the cultivation of hybridoma cells in Basket Spinner. The cells were immobilized within the Fibra-Cel^® ^disks. For comparison, two Basket Spinners were used in parallel, one of them was packed with 5 gm of Fibra-Cel^® ^disks, and the other was used as a control for the cultivation of free cells. Samples were daily collected throughout the cultivation for the determination of cell viability using Trypan blue exclusion method. Glucose/lactate concentrations were determined using automatic glucose/lactate analyzer. The concentration of MAb was determined by direct ELISA assay.

## Results

### Determination of MAb specificity

Secondary fluorescence antibodies bounded to the produced antibody which in turn is bound to CD3 positive lymphocytes (T-cells) showed a percentage of CD3 positive lymphocytes of 76.68%. This was proved using indirect immunofluorescence staining of healthy volunteer T lymphocytes from peripheral blood. Forward scatter (FSC) versus side scatter (SSC) can allow for the differentiation of blood cells in a heterogeneous cell population.

When the "gated" cells were analyzed for their emitted fluorescence upon stimulation by the laser beam, high fluorescence is produced from the cells that react with FITC- anti-mouse specific antibody which reflects CD3 antibody content in the added culture supernatant. Histogram statistics showed that there were 2513 events inside the gated lymphocytes; the percentage of lymphocytes that were CD3 positive was 76.68%.

### Continuous production of MAb by the cultivation of hybridoma cells in Basket spinner

**Figure 1:**
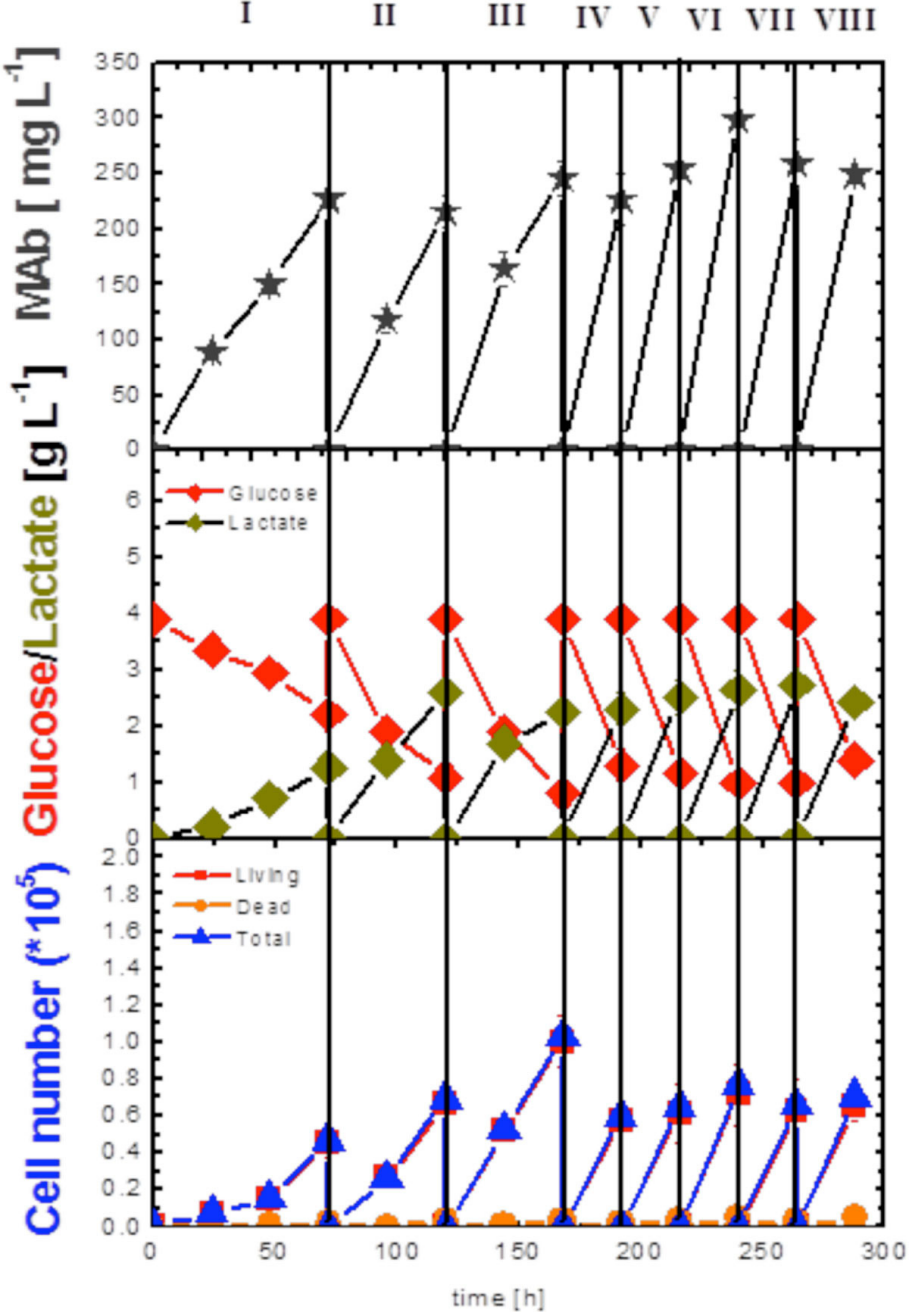
**Kinetics of cell growth, metabolism, and MAb production during cultivation of hybridoma cells in packed Basket Spinner**.

In this work two Basket Spinners were used in parallel, one of them was packed with 5 gm of Fibra-Cel disks (Figure 1), and the other was used as control without packing (free living cells). For the free Basket Spinner, the growth and viability of the hybridoma cells as well as their metabolic activities and mAb productivity were determined after 120 h. Viable cell concentration increased only during the first 72 h of cultivation reaching 9.2 × 10^5 ^Cells mL^-1^. On the other hand, mAb production reached its maximum concentration of 206.5 mg L^-1 ^also at 72 h. For the immobilized Basket Spinner, the growth and viability of the hybridoma cells as well as their metabolic activities and mAb productivity were determined for 288 h. The Culture medium was perfused through the bed to supply cells with nutrients. This allowed the spinner to run as repeated batch, enabling long term cultivation of cells. The number of viable, and dead cells determined over the 12 days of the cultivation corresponded to the cells detached from Fibra-CelR disks and does not reflect the actual cell number. On the other hand, the mAb titer increased in each batch reaching its maximum concentration of 298.5 mg L^-1 ^at batch number VI (after 216 h of cell inoculation).

It was found that the rates of glucose consumption and lactate production increased for each batch where the medium was changed once after the first 72 h and then the batch time was further reduced to only 48 h in the subsequent batches, then once each 24 h over the remaining 12 days of the cultivation period. The maximum production of lactate was 2.74 g L^-1 ^occurred at batch number VII (after 240 h).

Upon comparing at 72 h of cultivation, it was found that the produced mAb in case of the immobilized Basket Spinner was higher than that produced in case of the free Basket Spinner, however, the rate of glucose consumption and lactate production at the same time interval for the former was lower than the later (2.2, 1.825 g L^-1 ^for glucose and 1.27, 2.075 g L^-1 ^for lactate, respectively).

## Conclusion

The results obtained revealed that upon using flow cytometry and the fluorochrome-conjugated secondary antibody attached specifically to MAb present in the supernatant from the cells adapted to serum free medium succeeded in sorting 76.8% of the gated cells (lymphocytes). This confirmed the binding of MAb of the adapted cells to CD3 positive lymphocytes. Which means that, stable hybridoma cells adapted to grow in serum free medium (SMIF-6) were successfully obtained. It was also observed upon using the backed spinner basket, the MAb titer increased in each successive batch to reach to 298.5 mg L^-1 ^after 216 h. This might be due to the protection of the cells against shear stress and air/O_2 _sparging by their immobilization on the microcarriers, promoting the use of serum- or protein-free medium. Moreover, the microcarrier is designed to ensure sufficient nutrient supply and also to remove toxic metabolites. On the other hand, the rate of glucose consumption and lactate production increased for each repeated batch. This explains why the decrease in the batch period. This indicated the good physiological state of the cells.

